# PepMapViz: a versatile toolkit for peptide mapping, visualization, and comparative exploration

**DOI:** 10.1093/bioinformatics/btaf404

**Published:** 2025-07-15

**Authors:** Zhenru Zhou, Qui T Phung, Corey E Bakalarski

**Affiliations:** Department of Proteomic and Genomic Technologies, Genentech, Inc., South San Francisco, CA 94080, United States; Department of Proteomic and Genomic Technologies, Genentech, Inc., South San Francisco, CA 94080, United States; Department of Proteomic and Genomic Technologies, Genentech, Inc., South San Francisco, CA 94080, United States; Computational Catalysts, Genentech, Inc., South San Francisco, CA 94080, United States

## Abstract

**Summary:**

PepMapViz is a versatile R package that provides flexible peptide mapping and visualization capabilities. PepMapViz can import peptide data output from multiple popular mass spectrometry analysis tools, map peptides to their parent protein sequences, highlight protein domains and modifications, and enable comparative visualization across multiple experimental conditions. Beyond enabling visualization of MHC-presented peptide clusters in different antibody regions to predict potential immunogenicity of antibody-based therapies, PepMapViz can also aid in the visualization of cross-software mass spectrometry results at the peptide level for specific proteins, domain details in a linearized format, and post-translational modification coverage across different experimental conditions.

**Availability and implementation:**

PepMapViz is freely available on GitHub at https://github.com/Genentech/PepMapViz and on CRAN. The package is implemented in R and includes documentation and example datasets.

## 1 Introduction

Peptides play critical roles in diverse biological processes, from cellular signaling to defense mechanisms, and are of great interest as potential biomarkers and therapeutic agents (Hellinger *et al.* 2023). Mass spectrometry-based proteomics has become an essential tool for studying peptides and proteins, with many software systems available for the identification and quantification of peptides from spectral data. However, these tools predominantly concentrate on finding peptide spectrum matches (PSMs), and as such, providing visualization capabilities is often beyond their scope. For instance, many peptide search engines, such as Comet and MSFragger (Eng *et al.* 2013, Kong *et al.* 2017), do not provide built-in visualization capabilities, instead reporting data in text-based tabular reports. While amenable to high-throughput processing, data generated by these systems require further processing and visualization using additional tools to probe the underlying biological hypotheses being tested, especially for visualization of sequence-level features on specific proteins. Integrated pipelines, including the Trans Proteomic Pipeline (TPP), Fragpipe, Spectronaut and PEAKS Online, do offer limited peptide-level visualization within their data analysis platforms, but focus mostly on quality control of the analysis (Ma *et al.* 2003, Bruderer *et al.* 2015). Peptide-level visualization tools like pViz (Sauteraud* et al. * 2014) and Protter (Omasits *et al.* 2014) can enable interactive protein feature visualization and integration with experimental proteomic data, as can AlphaMap (Voytik *et al.* 2022), an open-source Python package for visual annotation of proteomics data with sequence-specific knowledge. However, these tools are limited in their ability to summarize multi-dataset results and facilitate comparative analyses, instead focusing on visualization of individual proteins and their features. Additionally, while some tools like AlphaMap offer flexibility in data input formats, many visualization tools are optimized for specific data formats or analysis platforms, creating challenges for researchers attempting to perform comparative analyses across outputs generated by different proteomics software systems.

PepMapViz enhances the usability of proteomics search engines by providing consistent and high-quality visualization of peptide and post-translational modification (PTM) mapping, without the need for specialized or expensive commercial visualization solutions. While advanced commercial platforms may offer rich visualization options, PepMapViz achieves comparable flexibility and publication-ready graphics within an open-source framework. The tool accepts outputs from a broad range of mass spectrometry software tools—including PEAKS, Spectronaut, MSFragger, Comet, MaxQuant, DIA-NN, and Skyline—as well as standard formats such as mzTab and mzIdentML, ensuring compatibility across most proteomics workflows. Beyond peptide-to-protein sequence display, it easily generates peptide plots that capture entire protein sequences in linearized format that are further augmented with protein domain and region details. Users can adjust the level of magnification, present each peptide distinctly, or opt for a collapsed version of peptides under the same condition, color-coded to reflect the peptide count, peak intensity or area mapping at each position. This flexibility enriches the understanding of the peptide spatial distribution and their functional implications, suitable for both iterative analysis of the results and for their final publication. Furthermore, PepMapViz includes an R Shiny application, providing an interactive graphical interface that further streamlines exploration, customization, and interpretation of peptide mapping results for users of all backgrounds.

A key feature of PepMapViz is its capacity to display various conditions with highlights of protein domain and region data within a single plot. This combined view fosters a deeper understanding of condition-specific patterns and trends, offering insights into the relationship between peptide distribution and experimental contexts. For instance, PepMapViz can cluster immunopeptides observed across different donors in MHC-associated peptide proteomics (MAPPs) analysis of protein or antibody drugs to assess potential immunogenic epitopes. This visual clustering across a large panel of donors and biotherapeutics is a key feature that facilitates immunogenicity de-risking of drug biotherapeutics of MAPPs data (Qin *et al.* 2025, Tsai *et al.* 2024). While there are existing MAPPs data processing pipelines, such as dataMAPPs (Steiner *et al.* 2020) that include visualization functions, they lack the flexibility to choose between intensity or PSM for color-coding peptides, do not support the visualization of post-translational modifications (PTMs), and are often compatible with only a single identification algorithm.

## 2 Features and implementation

**Figure 1.**
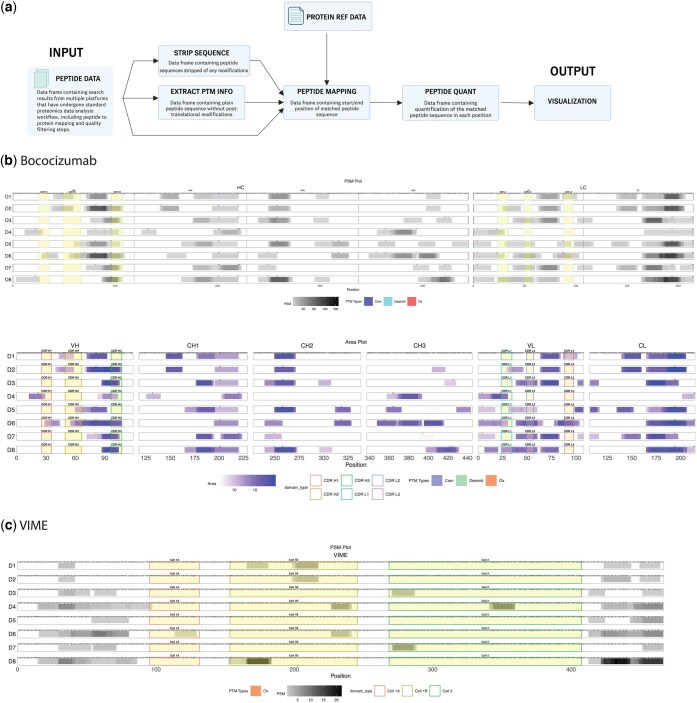
Workflow and example visualization of MAPPs results. (a) Workflow of PepMapViz. (b) Heatmap display of peptides detected across eight human donors for the antibody Bococizumab. Two heatmaps are shown for each antibody region. The top heatmap is colored according to the number of peptide spectrum matches (PSMs, greyscale gradient). The heavy chain (HC) and light chain (LC) antibody domains are separated, and all regions—variable region heavy chain (VH), constant region heavy chain 1 (CH1), constant region heavy chain 2 (CH2), constant region heavy chain 3 (CH3), variable region light chain (VL), and constant region light chain (CL)—and complementarity-determining region (CDR) boundaries (CDR H1, H2, H3, CDR L1, L2, L3) are labeled. The bottom heatmap is colored by peptide intensity (white to blue gradient). Different antibody domains—including variable region heavy chain (VH), constant region heavy chain 1 (CH1), constant region heavy chain 2 (CH2), constant region heavy chain 3 (CH3), variable region light chain (VL), and constant region light chain (CL)—are separated and labeled. Complementarity-determining region (CDR) boundaries are highlighted with distinct colors. Post-translational modifications (PTMs) are annotated with tick marks using predefined colors above the PSM heatmap bars, corresponding to the position of modified residue(s). (c) Heatmap of peptides detected across 8 human donors for protein Vimentin (VIME). Heatmaps are colored according to the number of peptide spectrum matches (PSMs) present in the region. Different protein domains, including Coil 1A, Coil 1B and Coil 2 are highlighted. PTMs are annotated with tick marks using predefined colors above the PSM heat map bars corresponding to the position of modified residue(s) as in (B).

PepMapViz is implemented as an R package, leveraging the data manipulation capabilities of data.table and the plotting flexibility of ggplot2. The modular design (Fig. 1a) allows users to customize each step of the analysis pipeline beyond the default options provided by the package if desired. In addition, PepMapViz includes an interactive R Shiny application, enabling users to explore and visualize their data through a user-friendly graphical interface without requiring programming experience.

### 2.1 Read files

The package is designed to process results from various mass spectrometry software outputs that have undergone a standard proteomics data analysis workflow, including quality control, normalization, and data transformation steps. PepMapViz supports text-based summarized formats expressed as TXT, CSV, or TSV, as well as standardized formats such as mzTab and mzIdentML. The currently supported mass spectrometry software includes PEAKS, Spectronaut, MSFragger, Comet, DIA-NN, Skyline, and MaxQuant. Additionally, it facilitates comparative analysis across different experimental conditions by enabling the simultaneous reading of multiple files. This function reads these files and creates a dataframe for streamlined data manipulation and analysis.

### 2.2 Strip sequences

This step processes outputs from multiple platforms, considering the varying ways different software present peptide sequences, including the presence or absence of flanking sequences and the representation of modification locations and types. It converts a dataframe containing peptide sequences with modifications into a new dataframe with plain peptide sequences, stripped of any modifications, ensuring consistency across diverse data formats. This function is optional, as some platforms already provide results with stripped sequences.

### 2.3 Extract post-translational modification information

This optional function handles outputs from multiple platforms, starting with a dataframe that includes a column of modified peptide sequences along with detailed PTM information. This step transforms the data into a new dataframe containing plain peptide sequences and their associated PTM details. If a sequence has multiple PTMs, each PTM is assigned a separate row in the result, and an additional column indicates if there are multiple rows due to multiple PTMs, ensuring that the peptide match count remains unchanged. Additionally, a table of PTM information can be manually added to control the desired annotation of PTMs in the generated output, providing further customization.

### 2.4 Peptide mapping

This function maps peptide sequences from the input dataframe with the provided target sequences. To address ambiguous or degenerate single-letter residue codes and those indistinguishable by mass spectrometry from the target sequence, we begin by decomposing peptides into their constituent amino acids and generating a set of potential amino acids for each position. For example, the sequence “NDEQIL” is converted to “[NBX][DBX][EZX][QZX][ILX][ILX],” allowing for all possible combinations to be mapped. While PepMapViz natively handles single-residue degeneracy, it can also be paired with the output of external tools such as Stitch (Schulte and Snijder 2024) to resolve larger mass coincidence ambiguities and produce more accurate antibody sequences for mapping. The output of this peptide mapping step includes the start and end positions of the mapped sequences along with a dataframe containing detailed information about these mapping positions. The provided target sequence can also include additional information, such as protein regions or domains.

### 2.5 Quantify matched peptide sequences

PepMapViz offers flexible options for the roll-up of quantitative results, including peptide spectral match (PSM) counts, intensity, and peak area-based quantification, allowing the representation of peptide abundance in various ways. For PSM counts, each position is quantified by the number of peptides spanning that position. For intensity or peak area-based quantification, the intensity or peak area of unique peptides spanning that position is summed. The quantification of each position is subsequently used to color-code the peptide heatmap in the visualization step.

### 2.6 Visualization

Once preprocessing of the results is complete, the core of PepMapViz is its ability to generate customizable plots depicting peptide coverage across linearized protein sequences. Key visualization features include: (i) the flexibility to summarize data by different features of sequences and/or experimental conditions; (ii) color-coding of peptides based on counts or quantitative values, enhancing visual differentiation; (iii) the ability to highlight protein domains, regions, and mutations with manually specified colors for better contextual understanding; (iv) annotation of post-translational modifications to provide insights into protein modifications; and (v) enabling comparative visualization through a side-by-side comparison of peptide coverage across multiple experimental conditions, facilitating deeper biological insights.

### 2.7 Interactive visualization with Shiny

To further enhance user experience, PepMapViz includes an R Shiny application that provides an interactive interface for visualizing peptide coverage maps. The Shiny app allows users to upload their processed data, customize visualization parameters (such as color schemes, domain highlighting, and annotation options), and explore results dynamically without writing code. Plots are generated using ggplot2, ensuring high-quality and customizable visualizations. Users can interactively filter and compare peptide coverage across different proteins or experimental conditions and export publication-ready figures.

Beyond visualization, PepMapViz can facilitate statistical analyses as part of a broader workflow. The Shiny app allows users to export tabular data in formats compatible with statistical software, enabling researchers to conduct post-hoc quantitative or statistical analyses on selected regions of interest where sufficient signal exists. By combining its visualization functionality with the ability to integrate downstream statistical tools, PepMapViz supports a comprehensive approach to data analysis that balances qualitative exploration with quantitative validation.

PepMapViz facilitates rapid data exploration and interpretation, making it immediately accessible to both computational and experimental researchers. For a detailed explanation of the R Shiny app workflow, refer to Fig. 1, available as supplementary data at *Bioinformatics* online, which outlines the sequential steps and functionalities within the application.

## 3 Example application

To demonstrate the utility of PepMapViz, we present an example of its use analyzing MHC-associated peptide proteomics (MAPPs) data for immunogenicity assessment of biotherapeutic proteins. Previous studies have shown that the number of presented peptide sequence regions and T cell response rates in T-cell activation assay are correlated with the clinical immunogenicity of monoclonal antibodies (Karle *et al.* 2016, Rombach-Riegraf *et al.* 2014). Molecules with lower clinical anti-drug antibody (ADA) incidence rate tend to have fewer presented sequence regions and lower T cell response rates, while more immunogenic candidates exhibit elevated numbers of presented peptide sequence regions (Lee *et al.* 2023). By quantifying the number and distribution of these peptide sequence regions across different donor samples, researchers can gain critical insights into the potential immunogenicity of biotherapeutic candidates (Karle, 2020, Rombach-Riegraf *et al.* 2014).

PepMapViz is well-suited for this application, as it enables the visualization and comparative analysis of immunopeptides identified from MAPPs experiments across multiple donors. Figure 1b demonstrates the power of PepMapViz in this context, showing the peptide heatmaps for one biotherapeutic candidate: monospecific anti-proprotein convertase subtilisin/kexin type nine (PCSK9) antibody, bococizumab, which exhibited a high clinical ADA incidence rate of 48% in multiple Phase 3 clinical trials (Ridker *et al.* 2017). This visual comparison of mapped peptides for each donor demonstrates PepMapViz’s ability to identify potential immunogenic T cell epitopes in biotherapeutics, providing critical information to guide the development of less immunogenic candidates.

Additionally, Fig. 1c illustrates the peptide heatmap for the human protein Vimentin (VIME), demonstrating that PepMapViz can be adapted for broader applications beyond antibody sequences. This example underscores the versatility of PepMapViz in mapping and visualizing peptides for any protein, thus extending its utility across various proteomics studies.

To further illustrate the utility of PepMapViz in immunogenicity assessment, two specific applications are highlighted. First, in an immunogenicity risk assessment of knobs-into-holes (KIH) bispecific IgG1 antibodies, a panel of mono- and bispecific IgG1s—including parental, engineered, and benchmark antibodies—were evaluated using complementary *in silico* T cell epitope prediction, dendritic cell internalization, T cell proliferation, and MHC-associated peptide proteomics (MAPPs) assays. The resulting peptide presentation and epitope landscape, visualized with PepMapViz, revealed that KIH mutations and Fab mutations used for efficient *in vivo* assembly did not confer major immunogenicity risks, as evidenced by *ex vivo* and *in vitro* readouts comparable to low-immunogenicity controls such as Avastin and Herceptin (Tsai *et al.* 2024).

Specifically, PepMapViz enabled the identification and quantification of peptide clusters, which are crucial for assessing immunogenicity risk. In this study, fewer peptide clusters were observed for the low ADA benchmark Avastin (*n* = 16) compared to high ADA benchmarks like bococizumab (*n* = 31) and ATR-107 (*n* = 28). Despite similar levels of amino acid side chain modifications, research-grade antibodies exhibited greater peptide spectrum matches (PSM) and sequence coverage, especially in variable domains. Additionally, the study assessed KIH mutations, finding that only one hole mutation (Y407V) was present in MAPPs peptides for the Tras/Bev 2 cell bispecific antibody—a mutation also observed in onartuzumab, a molecule associated with low clinical ADA risk. This indicates that the presence of the Y407V mutation does not necessarily lead to high ADA risk.

Moreover, comparative analysis showed a similar number of peptide clusters in various bispecific antibodies, with unexpected findings such as fewer clusters in the arm expected to be more immunogenic. For instance, the Boco/Bev bispecific antibody had comparable cluster numbers for bococizumab and bevacizumab arms. Such insights, facilitated by PepMapViz, underscore its role in systematic immunogenicity risk assessment, providing actionable insights for candidate selection and regulatory submissions.

This integrated approach, and the comparative heatmaps generated by PepMapViz, facilitate systematic immunogenicity risk assessment of bispecific antibodies and engineering variants, providing actionable insights for candidate selection and regulatory submissions.

In a second example, PepMapViz allows for visualization of the effects of single point mutations. A single amino acid substitution or engineered change in a therapeutic protein (such as FLT3L-Fc in this study) can significantly increase immunogenicity risk by creating new T cell epitopes (Qin *et al.* 2025). PepMapViz enables researchers to directly map and visualize peptide coverage and PTM landscapes around such mutations, facilitating identification and monitoring of newly generated immunogenic regions—an essential capability for protein engineering and preclinical safety assessment. These examples underscore the value of PepMapViz for immunogenicity risk assessment of complex antibody formats and engineered proteins, supporting rational biotherapeutic design and development.

## 4 Conclusion

PepMapViz offers a comprehensive, flexible toolkit for peptide mapping and visualization, addressing limitations in existing software, and provides a user-friendly solution to researchers working with peptide data. Its ability to integrate data from multiple sources, provide customizable visualizations, and enable comparative visual analysis makes it a valuable tool for researchers working with peptide data across various experimental contexts. These capabilities empower researchers to uncover patterns, identify condition-specific trends, and elucidate the relationship between peptide behavior and biological context.

We demonstrated the utility of PepMapViz in the context of evaluating the immunogenicity potential of biotherapeutic proteins. By leveraging the tool’s peptide clustering and visualization features, researchers can quantify the number and distribution of MHC-associated peptide clusters across different donor samples, providing valuable insights into the immunogenic epitopes within a protein sequence. This information can guide the optimization of biotherapeutic candidates, ultimately improving their chances of success in clinical development. Additionally, PepMapViz can be adapted for broader applications, such as visualizing potentially druggable cysteines proteome-wide in target proteins to identify potential drug binding sites (Boatner *et al.* 2023). By seamlessly integrating data from multiple mass spectrometry platforms and providing versatile visualization and comparative analysis capabilities, PepMapViz empowers researchers to gain deeper understanding of the role of peptides in biology and accelerate the development of peptide-based therapeutics and biomarkers.

## Supplementary Material

btaf404_Supplementary_Data

## Data Availability

The data underlying this article are available in the repository on GitHub: https://github.com/Genentech/PepMapViz/tree/master/inst/extdata.
