# Supplementary material for: PepMapViz: a versatile toolkit for peptide mapping, visualization, and comparative exploration
Source: Bioinformatics. 2025 Jul 15;41(7):btaf404. doi: 10.1093/bioinformatics/btaf404 (PMC12303863; doi:10.1093/bioinformatics/btaf404)
Supplement: btaf404_Supplementary_Data [file btaf404_supplementary_data.zip › 2.pdf]

PepMapViz: A Versatile Toolkit for Peptide Mapping, Visualization, and Comparative Exploration

Zhenru Zhou[1], Qui T Phung[1], Corey E Bakalarski[1,2]

Departments of [1]Proteomic and Genomic Technologies and [2]Computational Catalysts, Genentech, Inc., 1 DNA Way, South San Francisco, CA, USA 94080

Supporting Information

- Overview of the PepMapViz R Shiny Application Workflow for Interactive Peptide Visualization (Figure S1)

Supplementary Figure S1. *Overview of the PepMapViz R Shiny application workflow for interactive peptide visualization.* (A) Overview of R shiny application workflow. The application consists of several sequential steps: (B) Select Input Proteomics Files: Users can select multiple input files in various formats (CSV, TSV, TXT, mzID, mzTab) and optionally upload metadata files to merge. The interface allows specifying merge columns for integration. (C) Strip the Sequence (Optional): This step involves choosing the data type and defining columns for stripping sequences. Users can perform the operation by clicking "Strip Sequences". (D) Extract Modifications (Optional): Users select the data type for modification extraction, specify relevant columns, and annotate PTM information using an editable PTM table. The modifications are extracted upon user command. (E) Match Peptide Sequences: Users specify columns for matching peptide sequences against provided data. An editable sequence table aids in the matching process, with options to upload additional sequence data and set matching parameters. There are also options to input additional columns to match in addition to matching peptide sequences and add sequence length filtering. (F) Peptide Quantification: The application provides options for peptide quantification using methods like PSM and Area. Users can customize matching columns and distinct columns and choose to include PTM and replicate information. (G) Merge with Region Information (Optional): Region data can be uploaded and merged with the peptide data using an editable region table. Users define match columns and position columns for the merging process. (H) Plot Peptides: Users can configure plot settings to visualize peptides across sequences, including domain and PTM settings. The application supports advanced customization of plot appearance and legend configuration. The application interface allows users to preview data outputs at each processing step and download results. Visualization of peptide plots is facilitated, with options to download in chosen file formats (PDF or PNG).

Supplementary Figure S1. Overview of the PepMapViz R Shiny application workflow for interactive peptide visualization

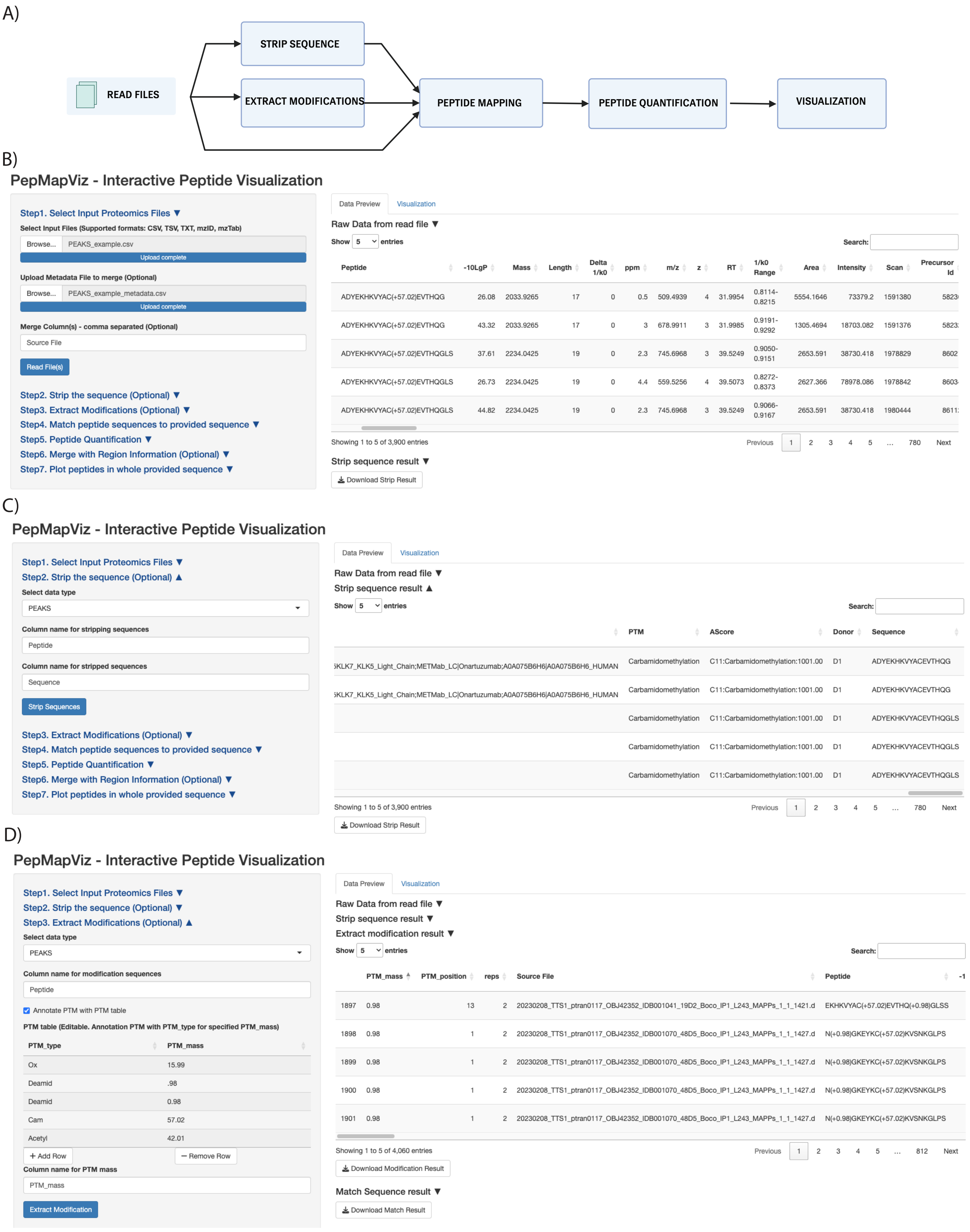

E)

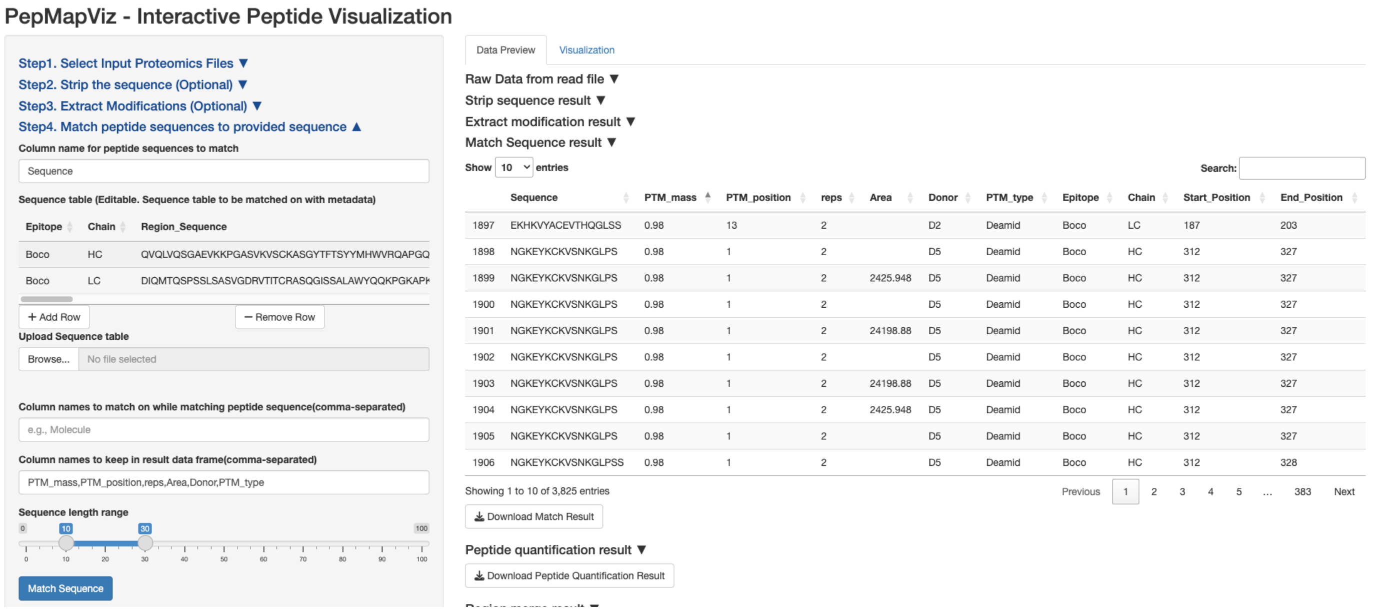
PepMapViz - Interactive Peptide Visualization
Step1. Select Input Proteomics Files ▼
Step2. Strip the sequence (Optional) ▼
Step3. Extract Modifications (Optional) ▼
Step4. Match peptide sequences to provided sequence ▲
Column name for peptide sequences to match
Sequence
Sequence table (Editable. Sequence table to be matched on with metadata)
Epitope Chain Region_Sequence
Boco HC QVQLVQSGAEVKKPGASVKVSCKASGYTFTSYYMHWVRQAPGQ
Boco LC DIQMTQSPSSLSASVGDRVTITCRASQGISSALAWYQQKPGKAPK
+ Add Row
— Remove Row
Upload Sequence table
Browse...
No file selected
Column names to match on while matching peptide sequence(comma-separated)
e.g., Molecule
Column names to keep in result data frame(comma-separated)
PTM_mass,PTM_position,reps,Area,Donor,PTM_type
Sequence length range
0 10 30 100
0 10 20 30 40 50 60 70 80 90 100
Match Sequence
Data Preview
Visualization
Raw Data from read file ▼
Strip sequence result ▼
Extract modification result ▼
Match Sequence result ▼
Show 10 entries
Search:
Sequence PTM_mass PTM_position reps Area Donor PTM_type Epitope Chain Start_Position End_Position
1897 EKHKVYACEVTHQGLSS 0.98 13 2 D2 Deamid Boco LC 187 203
1898 NGKEYKCKVSNKGLPS 0.98 1 2 D5 Deamid Boco HC 312 327
1899 NGKEYKCKVSNKGLPS 0.98 1 2 2425.948 D5 Deamid Boco HC 312 327
1900 NGKEYKCKVSNKGLPS 0.98 1 2 D5 Deamid Boco HC 312 327
1901 NGKEYKCKVSNKGLPS 0.98 1 2 24198.88 D5 Deamid Boco HC 312 327
1902 NGKEYKCKVSNKGLPS 0.98 1 2 D5 Deamid Boco HC 312 327
1903 NGKEYKCKVSNKGLPS 0.98 1 2 24198.88 D5 Deamid Boco HC 312 327
1904 NGKEYKCKVSNKGLPS 0.98 1 2 2425.948 D5 Deamid Boco HC 312 327
1905 NGKEYKCKVSNKGLPS 0.98 1 2 D5 Deamid Boco HC 312 327
1906 NGKEYKCKVSNKGLPSS 0.98 1 2 D5 Deamid Boco HC 312 328
Showing 1 to 10 of 3,825 entries
Previous 1 2 3 4 5 … 383 Next
Download Match Result
Peptide quantification result ▼
Download Peptide Quantification Result

F)

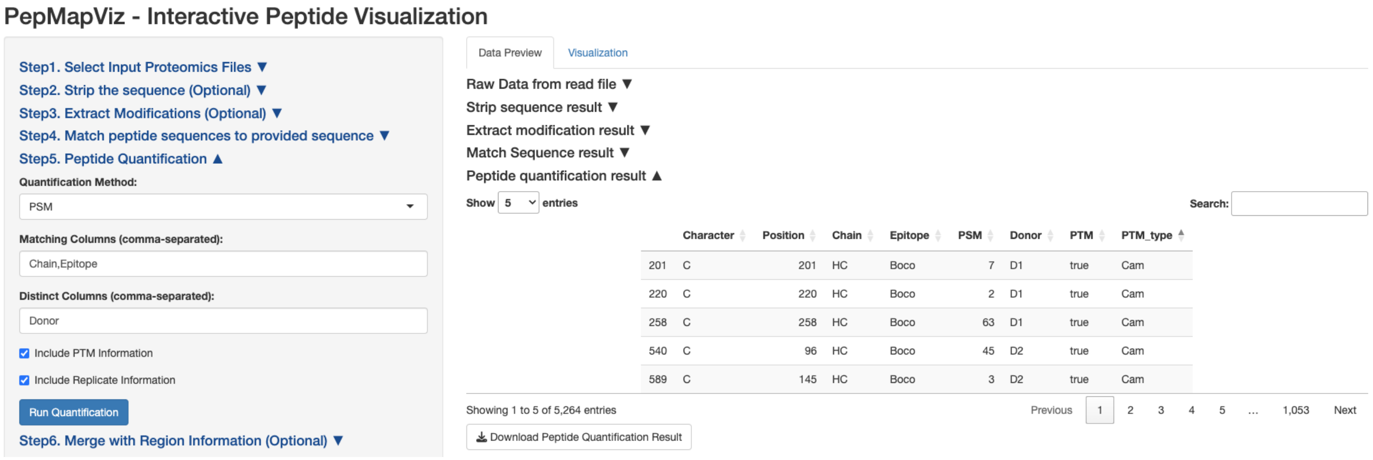
PepMapViz - Interactive Peptide Visualization
Step1. Select Input Proteomics Files ▼
Step2. Strip the sequence (Optional) ▼
Step3. Extract Modifications (Optional) ▼
Step4. Match peptide sequences to provided sequence ▼
Step5. Peptide Quantification ▲
Quantification Method:
PSM
Matching Columns (comma-separated):
Chain,Epitope
Distinct Columns (comma-separated):
Donor
Include PTM Information
Include Replicate Information
Run Quantification
Step6. Merge with Region Information (Optional) ▼
Data Preview
Visualization
Raw Data from read file ▼
Strip sequence result ▼
Extract modification result ▼
Match Sequence result ▼
Peptide quantification result ▲
Show 5 entries
Search:
Character Position Chain Epitope PSM Donor PTM PTM_type
201 C 201 HC Boco 7 D1 true Cam
220 C 220 HC Boco 2 D1 true Cam
258 C 258 HC Boco 63 D1 true Cam
540 C 96 HC Boco 45 D2 true Cam
589 C 145 HC Boco 3 D2 true Cam
Showing 1 to 5 of 5,264 entries
Previous 1 2 3 4 5 … 1,053 Next
Download Peptide Quantification Result

G)

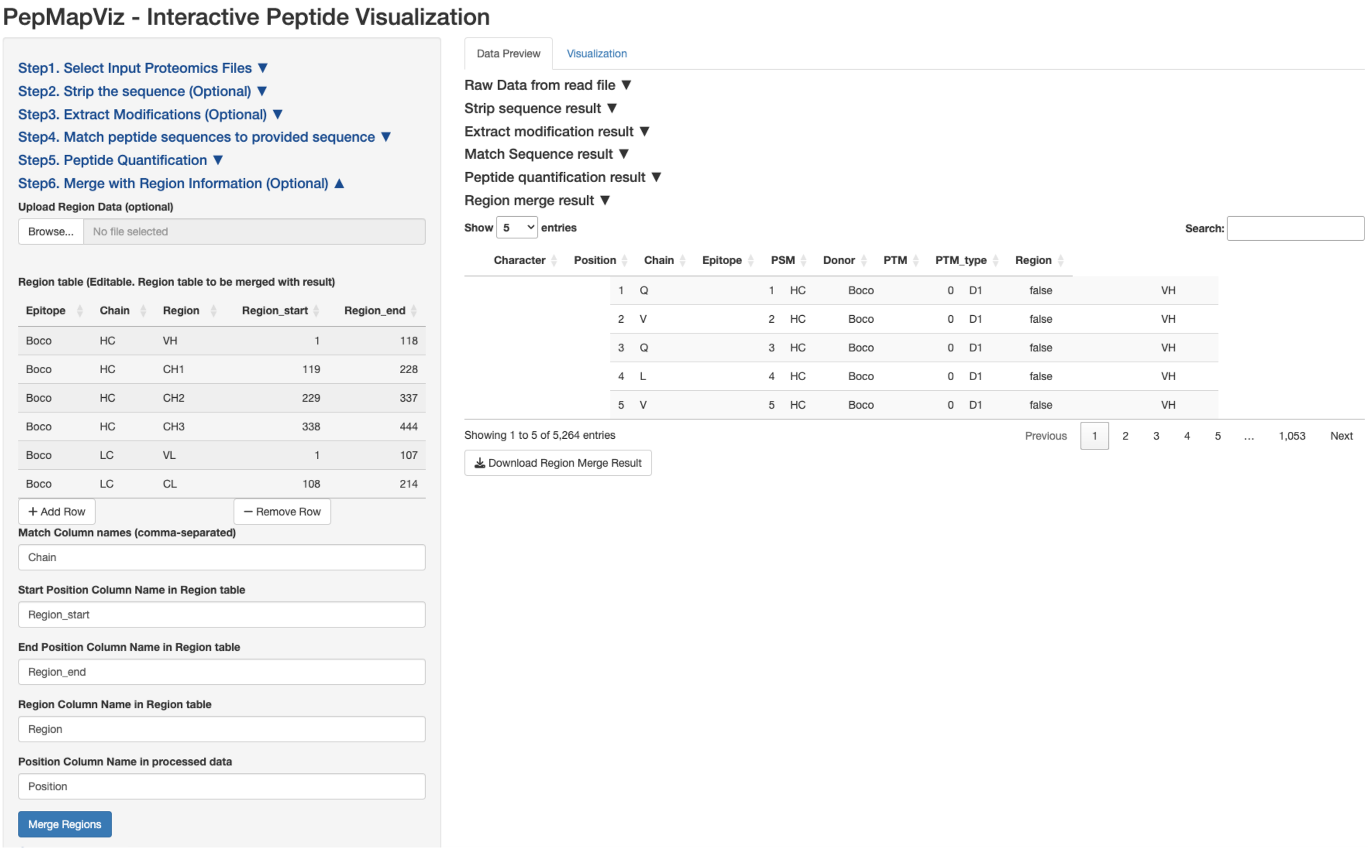
PepMapViz - Interactive Peptide Visualization
Step1. Select Input Proteomics Files ▼
Step2. Strip the sequence (Optional) ▼
Step3. Extract Modifications (Optional) ▼
Step4. Match peptide sequences to provided sequence ▼
Step5. Peptide Quantification ▼
Step6. Merge with Region Information (Optional) ▲
Upload Region Data (optional)
Browse...
No file selected
Region table (Editable. Region table to be merged with result)
Epitope Chain Region Region_start Region_end
Boco HC VH 1 118
Boco HC CH1 119 228
Boco HC CH2 229 337
Boco HC CH3 338 444
Boco LC VL 1 107
Boco LC CL 108 214
+ Add Row
− Remove Row
Match Column names (comma-separated)
Chain
Start Position Column Name in Region table
Region_start
End Position Column Name in Region table
Region_end
Region Column Name in Region table
Region
Position Column Name in processed data
Position
Merge Regions
Data Preview
Visualization
Raw Data from read file ▼
Strip sequence result ▼
Extract modification result ▼
Match Sequence result ▼
Peptide quantification result ▼
Region merge result ▼
Show 5 entries
Search:
Character Position Chain Epitope PSM Donor PTM PTM_type Region
1 Q 1 HC Boco 0 D1 false VH
2 V 2 HC Boco 0 D1 false VH
3 Q 3 HC Boco 0 D1 false VH
4 L 4 HC Boco 0 D1 false VH
5 V 5 HC Boco 0 D1 false VH
Showing 1 to 5 of 5,264 entries
Previous 1 2 3 4 5 … 1,053 Next
Download Region Merge Result

H)

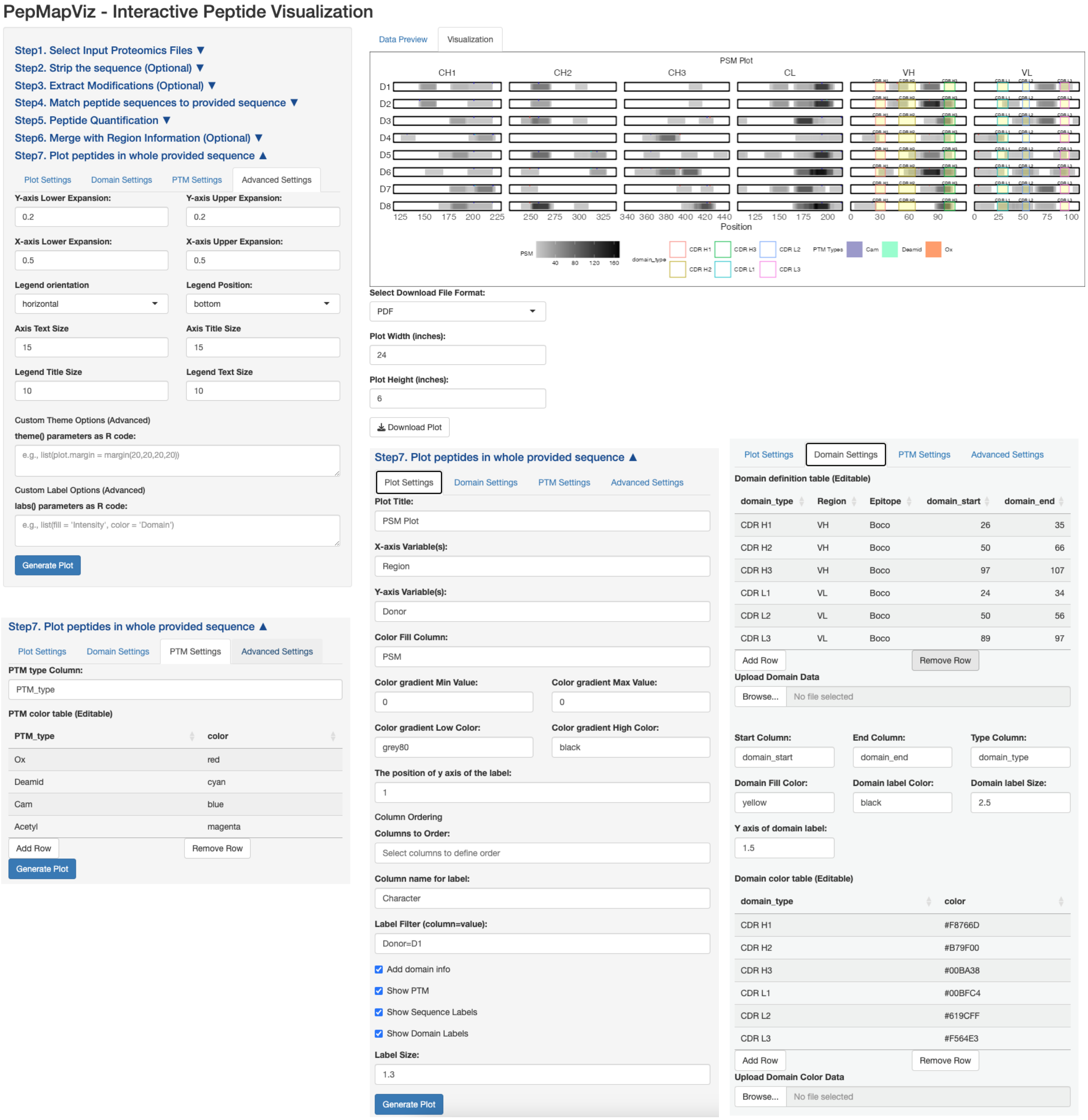
PepMapViz - Interactive Peptide Visualization
Step1. Select Input Proteomics Files ▼
Step2. Strip the sequence (Optional) ▼
Step3. Extract Modifications (Optional) ▼
Step4. Match peptide sequences to provided sequence ▼
Step5. Peptide Quantification ▼
Step6. Merge with Region Information (Optional) ▼
Step7. Plot peptides in whole provided sequence ▲
Plot Settings
Domain Settings
PTM Settings
Advanced Settings
Y-axis Lower Expansion:
0.2
Y-axis Upper Expansion:
0.2
X-axis Lower Expansion:
0.5
X-axis Upper Expansion:
0.5
Legend orientation
horizontal
Legend Position:
bottom
Axis Text Size
15
Axis Title Size
15
Legend Title Size
10
Legend Text Size
10
Custom Theme Options (Advanced)
theme() parameters as R code:
e.g., list(plot.margin = margin(20,20,20,20))
Custom Label Options (Advanced)
labs() parameters as R code:
e.g., list(fill = 'Intensity', color = 'Domain')
Generate Plot
Data Preview
Visualization
PSM Plot
CH1
CH2
CH3
CL
VH
VL
D1
D2
D3
D4
D5
D6
D7
D8
Position
PSM
domain_type
CDR H1
CDR H2
CDR H3
CDR L1
CDR L2
CDR L3
PTM Types
Cam
Deamid
Ox
Select Download File Format:
PDF
Plot Width (inches):
24
Plot Height (inches):
6
Download Plot
Step7. Plot peptides in whole provided sequence ▲
PTM type Column:
PTM_type
PTM color table (Editable)
PTM_type | color
Ox | red
Deamid | cyan
Cam | blue
Acetyl | magenta
Add Row
Remove Row
Generate Plot
Step7. Plot peptides in whole provided sequence ▲
Plot Title:
PSM Plot
X-axis Variable(s):
Region
Y-axis Variable(s):
Donor
Color Fill Column:
PSM
Color gradient Min Value:
0
Color gradient Max Value:
0
Color gradient Low Color:
grey80
Color gradient High Color:
black
The position of y axis of the label:
1
Column Ordering
Columns to Order:
Select columns to define order
Column name for label:
Character
Label Filter (column=value):
Donor=D1
Add domain info
Show PTM
Show Sequence Labels
Show Domain Labels
Label Size:
1.3
Generate Plot
Domain definition table (Editable)
domain_type | Region | Epitope | domain_start | domain_end
CDR H1 | VH | Boco | 26 | 35
CDR H2 | VH | Boco | 50 | 66
CDR H3 | VH | Boco | 97 | 107
CDR L1 | VL | Boco | 24 | 34
CDR L2 | VL | Boco | 50 | 56
CDR L3 | VL | Boco | 89 | 97
Add Row
Remove Row
Upload Domain Data
Browse...
No file selected
Start Column:
domain_start
End Column:
domain_end
Type Column:
domain_type
Domain Fill Color:
yellow
Domain label Color:
black
Domain label Size:
2.5
Y axis of domain label:
1.5
Domain color table (Editable)
domain_type | color
CDR H1 | #F8766D
CDR H2 | #B79F00
CDR H3 | #00BA38
CDR L1 | #00BFC4
CDR L2 | #619CFF
CDR L3 | #F564E3
Add Row
Remove Row
Upload Domain Color Data
Browse...
No file selected
