# Supplementary material for: PepMapViz: a versatile toolkit for peptide mapping, visualization, and comparative exploration
Source: Bioinformatics. 2025 Jul 15;41(7):btaf404. doi: 10.1093/bioinformatics/btaf404 (PMC12303863; doi:10.1093/bioinformatics/btaf404)

Supplementary Figure S1. Overview of the PepMapViz R Shiny application workflow for interactive peptide visualization

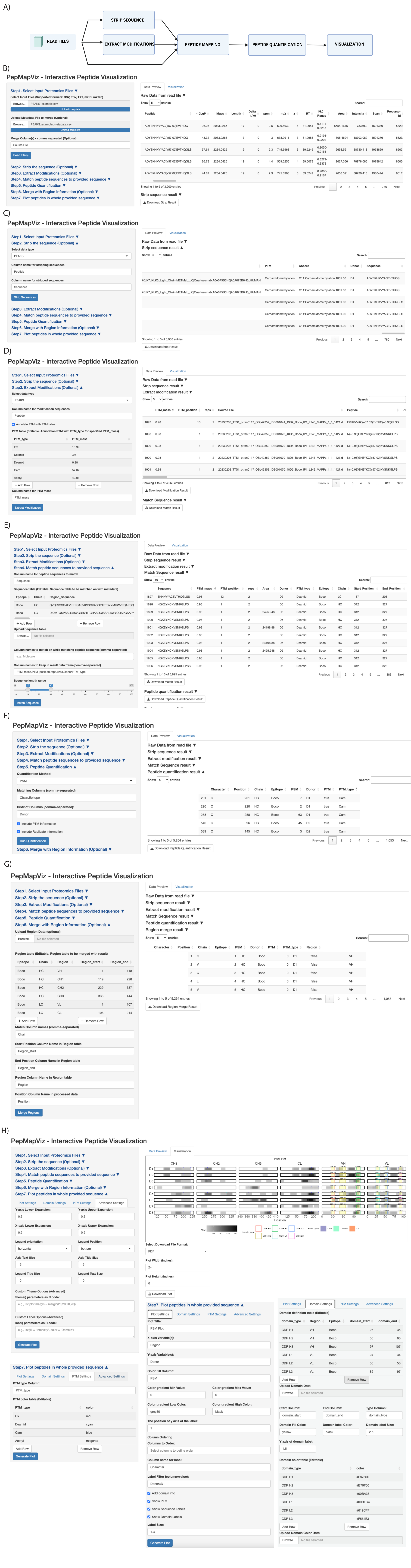

Supplement: btaf404_Supplementary_Data [file btaf404_supplementary_data.zip › PepMapViz_supple_figure.pdf]
